# Validity of the six-minute step test of free cadence in patients with chronic obstructive pulmonary disease

**DOI:** 10.1590/bjpt-rbf.2014.0041

**Published:** 2014

**Authors:** Bruna V. Pessoa, Juliano F. Arcuri, Ivana G. Labadessa, Joyce N. F. Costa, Anna C. Sentanin, Valéria A. Pires Di Lorenzo

**Affiliations:** 1 Laboratory of Spirometry and Respiratory Therapy, Universidade Federal de São Carlos (UFSCar), São Carlos, SP, Brazil; 2 Laboratory of Cardiorespiratory Physical Therapy, Universidade do Sagrado Coração (USC), Bauru, SP, Brazil

**Keywords:** COPD, physical therapy, validity, exercise test

## Abstract

**Objectives:**

to evaluate the concurrent validity of the six-minute step test (6MST) in assessing exercise capacity of COPD patients using the six-minute walk test (6MWT) as a gold-standard. The predictive validity of the 6MST was assessed to determine a cut-off point for identification of low exercise capacity.

**Method:**

thirty-two COPD patients (50-87 years old) with mild to very severe obstruction performed the 6MST and 6MWT twice.

**Results:**

Concurrent validity: a strong positive correlation (Pearson) between the number of ascents on the first (T1), second (T2) and the best of both (T1 or T2) tests during the 6MWT was observed. Although a moderate negative correlation with BODE index and FEV_1_ was found, it was considered insufficient to test the validity, therefore ROC curves were not applied. The predictive validity (ROC) of the 6MST to identify low physical capacity (compared with the 6MWT) using the performance of T1 or T2, or solely T1 was considered accurate, and the area under the curve was 0.8 (IC95% 0.62-0.98) and 0.85 (IC95% 0.70-0.99), respectively. To classify patients, the cut-off points of 86 and 78 steps were chosen, with both values showing 90% of sensitivity and specificity of 64% and 68% for T1 or T2, or solely T1, respectively.

**Conclusion:**

The number of steps on the 6MST was valid to verify exercise capacity in COPD patients and the cut-off point of 78 steps was able to identify patients with poor exercise tolerance. Values under this cut-off point are considered to identify patients with a poorer prognosis.

## Introduction

Chronic obstructive pulmonary disease (COPD) is characterized by decreased aerobic capacity^1^ and muscle strength^2^, culminating in loss of functionality and exercise intolerance. These changes are the most important consequences of this disease^3^, which negatively impacts the quality of life of patients^1^. Therefore, it is necessary to evaluate exercise tolerance in COPD patients through functional tests, since the tests are able to provide specific information concerning functional capacity and physiological adaptation during physical effort.

The maximal cardiopulmonary test is considered the gold-standard for assessing exercise tolerance because it evaluates functional capacity and abnormal responses of the musculoskeletal, cardiovascular and respiratory systems. In addition, it has been widely used in the prescription and monitoring of physical training in cardiac and pulmonary rehabilitation^4^ ^,^ ^5^. However, its use is limited in clinical practice due to the complexity of the equipment, high costs and the need for trained technicians^6^.

Therefore, alternative forms of assessment to the maximal test, such as the 6-minute walk test (6MWT) and the 6-minute step test (6MST) began to stand out for being practical and easy to perform in clinical settings^7^ ^,^ ^8^. The 6MWT is a test that has been used in different populations^9^ ^-^ ^13^ and is considered valid and reproducible. In addition, it is a good predictor of morbidity and mortality of patients with COPD^12^ ^,^ ^14^. In a recent and large cohort of 2110 COPD patients Spruit et al.^15^determined the distance of 334 m as a predictor of mortality. In a period of three years, 62.5 % of the patients with values below this performance level died, emphasizing the importance of the prognostic value. Despite the advantages presented in relation to the 6MWT, the physical space requirements for its implementation is sometimes a limiting factor^7^ ^,^ ^8^, especially in primary care. Thus, finding alternative solutions are important.

On the other hand, the 6MST consists of a self-paced test performed on a step with fixed dimensions and it is considered a simple and effective alternative because it requires less physical space in addition to being portable^8^ ^,^ ^16^.

Studies conducted for nearly 80 years have shown the importance of step tests in the evaluation of physical fitness of healthy individuals by detecting possible abnormalities in physiological responses^17^ ^,^ ^18^. Schnaider and Karsten^19^ observed that the performance of hospitalized patients with COPD when doing the 6MST showed good correlation with the 6MWT, suggesting that it could replace the 6MWT in a hospital environment. Machado et al.^20^ found significant correlations between oxygenation, fatigue of the lower limbs (LL) and diastolic blood pressure at the peak of these two tests and concluded that the 6MST could be an alternative to the 6MWT.

Recently, the 6MST was validated in patients with interstitial lung disease^8^. The study showed that it is reproducible in addition to it being safe and sensitive to oxygen desaturation induced by exercise, easy to use, economical and portable^8^. However, there are currently no guidelines for the use of 6MST in patients with COPD^21^ and the literature lacks concurrent validity and predictive criterion of this test in patients with COPD.

Therefore, the objectives of this study were to assess the concurrent validity of the number of steps on the 6MST in physical capacity of patients with COPD using the 6MWT as the gold-standard test, as well as to verify the predictive validity criterion of the 6MST by determining the cut-off value to identify patients with low exercise capacity and consequently a poor prognostic outcome.

## Method

### Study subjects

The present study is part of a major observational, prospective, cross-sectional study, conducted at the Laboratory of Spirometry and Respiratory Physical Therapy, Universidade Federal de São Carlos (UFSCar), São Carlos, SP, Brazil. The major study was designed to examine the clinimetric properties (validity and reliability) of the 6MST and 6MWT in three different populations (patients with COPD, young adults, and elderly). The study was registered in Clinical Trials. Gov. (NCT01298661). The present study specifically aimed to determine the validity of the 6MST in patients with COPD.

Thirty-four subjects ( patients), aged 50 to 87 years, of both sexes, and referred for care at the Special Unit of Respiratory Therapy - UFSCar - during the period June 2011 to July 2012, were included in the study. The participants were invited through posters in the university and neighbourhood, by local radio, television and newspapers. Participants were contacted by phone and were invited to participate in the study if they fit the inclusion criteria.

Inclusion criteria were as follows: clinical and spirometric diagnosis of COPD, obstruction ranging from mild to very severe^21^, clinically stable and with no history of infection or exacerbation of respiratory symptoms in the last three months preceding the study. The exclusion criteria were: patients with exacerbated lung disease, decompensated heart disease, neuromuscular, rheumatic and orthopedic diseases that prevented performance of the exercise tests, controlled hypertension without the use of beta-blockers, those who did not complete one of the tests and those with peripheral oxygen saturation (SpO_2_) below 80% of physical efforts.

All participants signed a consent form, the same used in the major study, which described all evaluations and analyses used in the present study. The major study was approved by the Research Ethics of UFSCAR (Protocol No. 009/2011).

### Experimental protocol

All patients were submitted to a two day assessment, with a minimum interval of 48 hours between them. On the first day, anthropometric characteristics, vital signs, medications used, family history, smoking habits, presence of cough and dyspnea were obtained. In addition, one of the tests, 6MST or 6MWT, with the order determined by lottery, were conducted. On the second day, body composition was assessed as well as the test that was not drawn on the first day.

### Six-minute walking test (6MWT)

The 6MWT was performed along a level corridor 30 meters long and 1.5 meters wide. Patients were instructed and encouraged to walk as far as possible in six minutes, using standard incentive phrases every minute^7^. The 6MWT was performed twice on the same day with an interval of 30 minutes between the tests. The distance covered in each test was used for analysis.

SpO_2_ (Nonin^(r)^, model 2500, Minneapolis, Mn, USA), heart rate (HR, Polar^(r)^ Vantage NVTM, Model 1901001, Kempele, Oulu, Finland), symptoms of dyspnea and fatigue of the lower limbs were verified by the Borg modified scale CR10^22^. Blood pressure (BP) was obtained at rest and immediately after testing.

### Six-minute step test (6MST)

The 6MST was carried out by two raters: one to control the test and the other to count the steps. A step (20 cm high with non-slip rubber surfave)^8^ was used as the test device. For better reproducibility, the 6MWT test followed the principles of the American Thoracic Society^7^, using standardized phrases every minute to encourage the patient. Patients were instructed to step up and down for six minutes, targeting the maximum number of steps (free cadence). Patients could alternate the lower limbs but upper limbs had no support and remained stationary alongside the body.

Like at the 6MWT, the SpO_2_, HR, BP and symptoms of dyspnea and fatigue of the LL^22^ were verified at rest, immediately after the tests and during the recovery period. The performance on the test (number of steps) was used for analysis.

### Assessment of body composition

Body composition was performed through a balance of bioelectrical impedance of bipolar technology (Tanita^(r)^ model CB-553, Illinois, USA). Analysis of body mass and muscle mass (MM)^23^ followed by body mass index (BMI) = body weight (kg) / height^2^ (m) and fat-free mass index (FFMI) = MM (kg) / height^2^ (m)^24^, were calculated. 

### Pulmonary function test

A pre and post-bronchodilator was conducted by the pulmonologist in order to verify the degree of obstruction, according to the guidelines of the American Thoracic Society/European Respiratory Society (ATS/ERS)^25^. The values obtained were compared to those expected for the Brazilian population^26^. 

### BODE index

Patients with COPD were assessed in all necessary measures to calculate the BODE index. This multidimensional index is composed by the BMI, the degree of airway obstruction (FEV1% expected post-bronchodilator)^21.26^, dyspnea (dyspnea scale from the Medical Research Council)^27^ and by the distance covered during the 6MWT^7^. Patients were scored according to the results obtained in the four variables (0-1 to BMI and 0-3 to FEV1, dyspnea and distance covered during the 6MWD)^28^. The BODE index may be divided into quartiles: quartile 1 is the score of 0-2, quartile 2 is the score of 3-4, quartile 3 is the score of 5-6 and quartile 4 is the score of 7-10^28^. The higher the score, the higher the probabilty of mortality of patients^28^.

### Statistical analysis

To calculate the sample size, the authors considered that it would be necessary to have a correlation of r=0.80 between the performances of the tests, using as the null hypothesis a correlation below r=0.5, so that the value of r would be considered moderate - assuming an α error of 5% and β error of 20%^29^. According to these data, the sample size was calculated to be 29 subjects, corresponding to a statistical power of 80%.

The normality of the data was verified using the Shapiro-Wilk test. Data were expressed as a mean and standard deviation for normally distributed data and as a median (interquartile range) for data not normally distributed. The level of confidence was set at 5%.

The validity of the 6MST was verified using the concurrent and predictive validity criteria. For concurrent validity, a Pearson correlation coefficient^30^ ^,^ ^31^ was conducted between the performances of the 6MWT and 6MST using the values of T1 (first test) or T2 (second test) obtained in both tests. Correlations were also conducted to verify the relationship between T1 on the 6MST with the better of the two tests (T1 or T2) of the 6MWT. To verify the relationship between performances on the 6MST and BODE index predictor of mortality, the Spearman correlation coefficient was used. For the classification of this coefficient, a criterion of r>0.7 would indicate that the instrument was valid^32^. 

For the predictive validity criteria, ROC curves were constructed to verify the sensitivity and specificity of the 6MST in classifying patients with COPD. The cut-off point was determined from the highest sensitivity and specificity for the best number of ascents on the step test of T1 or T2, and T1 only. Therefore, the area under the curve was used as a criterion to determine the validity of the test. If the test presented values below 0.7, they were considered to have low accuracy. Values above 0.7 but below 0.9 would be considered to be accurate for some purposes. Lastly, values over 0.9 would be considered highly accurate^33^. To verify whether the 6MST presented validity to classify patients with low physical capacity, the 6MWT was used as the reference test with a cut-off of 334 m^15^. According to Spruit et al.^15^, the cut-off value of 334 m is a predictor of mortality, since patients with COPD who had values below this cut-off died within a period of three years, emphasizing the importance of this prognostic value.

## Results

Thirty-four patients were initially included in the study, however two were excluded for not completing the assessment (abandoned the study). Of the 32 patients (24 male and eight female) included in the analysis, two had COPD grade I, nine grade II, 15 grade III and six stage IV^21^.

The sample characteristics are shown in Table 1. Table 2 shows the performance during the 6MWT and 6MST of the COPD patients evaluated.

**Table 1:** Anthropometric, spirometry and BODE index characteristics of the COPD patients.

Characteristics	COPD Patients (n=32)
Anthropometrics	
Age (years)	69±10
Body mass (kg)	67±12
Height (m)	1.6±0.1
BMI (kg/m^2^)	25±4
FFMI (kg/m^2^)	15.4±5.2
Body Fat (%)	24.0±12.1
Spirometry	
FVC (% pred)	62.7±19.3
FEV_1_ (%pred)	45.8±17.7
FEV_1_/FVC (%)	54.1±12.8
BODE Index	3 (1-4)

Data are expressed as mean ± standard deviation; median (interquartile range)

COPD = chronic obstructive pulmonary disease

BMI = body mass index

FFMI = fat-free mass index

FVC = forced vital capacity

FEV1 = forced expiratory volume in one second

FVC/FEV1 = FVC/FEV_1_ ratio

**BODE index:** = Body mass index, Obstruction, Dyspnea and Exercise capacity

**Table 2:** Performance of COPD patients on the six-minute step test and six-minute walk test.

COPD Patients (n=32)	6MST (steps)	6MWT (distance travelled, m)
T1	76.7±19.6	380.4±107.8
T2	82.4±20.7	391.0±94.0
T1 or T2	83.2±20.2	402.4±102.3

Data are expressed as mean ± standard deviation

COPD = chronic obstructive pulmonary disease

6MST = six minute step test

6MWT = six minute walk test

T1 and T2 = performance on the first and second 6MST or 6MWT, respectively

T1 or T2 = Best performance on the first or second 6MST or 6MWT, respectively

### Concurrent criterion validity

**Figure 1:**
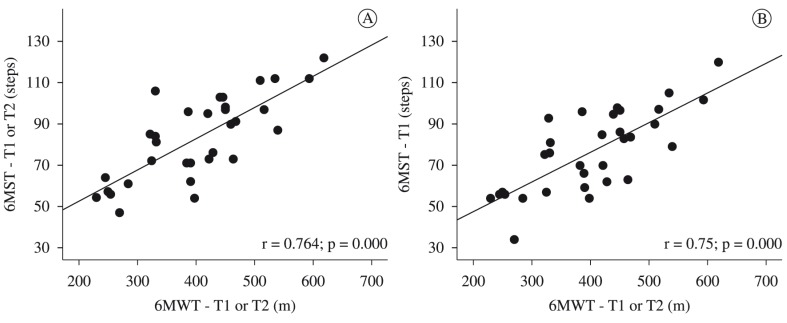
Relationship between performance on the 6MST and the 6MWT in patients with COPD. A = 6MST -T1 or T2 versus 6MWT -T1 or T2; B = 6MST -T1 versus 6MWT -T1 or T2. COPD = chronic obstructive pulmonary disease; 6MWT-T1 or T2 = Best performance on the first or second six-minute walk test; 6MST-T1= First six minute step test; 6MST-T1 or T2 = Best performance on the first or second six-minute step test.

The performance values at T1 and T2, and at T1 or T2 during the 6MST showed a strong and positive correlation that was statistically significant with the 6MWT in all tests, and may be considered valid to verify the physical capacity of patients with COPD (Table 3, Figure 1). In addition, we found a significant negative weak correlation between performance on the 6MST and BODE index, and a significant weak correlation between performances in 6MST and FEV1. However, these correlations were insufficient to characterize validity (Table 3).

**Table 3:** Correlations between performances on the 6MST and 6MWT in patients with COPD.

COPD Patients	6MST - T1	6MST -T2	6MST - T1 or T2
6MWT - T1	0.734*	0.777*	0.768*
6MWT - T2	0.733*	0.739*	0.739*
6MWT – T1 or T2	0.750*	0.766*	0.764*
FEV_1_	0.466*	0.376*	0.385*
BODE index	-0.503*	-0.457*	-0.451*

COPD = chronic obstructive pulmonary disease

6MST = six-minute step test

6MWT = six-minute walk test

T1 and T2 = performance on the first and second 6MST or 6MWT, respectively

T1 or T2 = Best performance on the first or second 6MST or 6MWT, respectively

FEV1 = forced expiratory volume in one second

**BODE index:** = Body mass index, Obstruction, Dyspnea and Exercise capacity index

* =p<0.05.

### Predictive criterion validity

**Figure 2:**
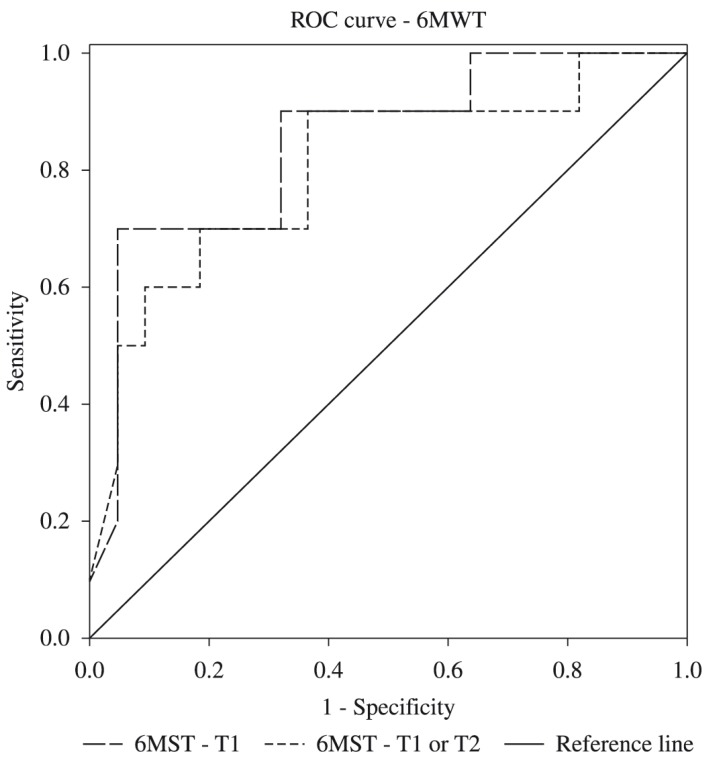
Sensitivity and specificity of the 6MST in predicting physical capacity in COPD patients, using as reference the 6MWT - T1 or T2, with a cut-off point of 334 m. 6MWT = Six-minute walk test; 6MWT-T1 or T2 = Best performance on the first or second six-minute walk test; 6MST-T1 = First six-minute step test; 6MST-T1 or T2 = Best performance on the first or second six-minute step test.

The criterion validity of the 6MST to identify low physical capacity (compared to the 6MWT), using the number of the steps at T1 or T2 or only in T1, was considered accurate for some purposes, with the area under the curve of 0.8 (95% CI 0.62 to 0.98) and 0.85 (95% CI 0.70 to 0.99), respectively. For the number of steps in T1 or T2, the cut-off was set at 86 steps, with sensitivity of 90% and specificity of 64% (Figure 2). However, for the number of steps at T1, the cut-off was set at 78 steps, with 90% sensitivity and 68% specificity to classify patients with COPD (Figure 2).

ROC curves were not plotted for the BODE index nor the FEV1, since construct validity was not found.

## Discussion

To the best of the authors knowledge, this is the first study performed to determine the predictive criterion validity of the 6MST to identify patients with low physical capacity and consequently poor prognosis in COPD. The number of steps in 6MST was valid in assessing exercise capacity in patients with COPD and could identify patients with low exercise tolerance and poor prognosis, so we suggest that this test may be used for this purpose.

The 6MST has shown to be clinically useful in estimating exercise tolerance in COPD^34^, and requires minimal physical space and only two evaluators to perform the test^8^ ^,^ ^16^. It is noteworthy that the 6MST, compared to 6MWT, uses specific muscle groups (quadriceps). In addition, according to Swinburn et al.^35^, the 6MST (to climb 15 steps/min at step height of 25 cm) provides greater metabolic and ventilatory stress compared to exercises on a cycle ergometer and during the 6MWT in patients with COPD. Thus, we suggest that the 6MST is a better test for accessing the level of physical exertion in these patients.

Pessoa et al.^36^ concluded that the 6MST, the first two minutes of the 6MST, and the two minute sit-to-stand test may be alternatives to assess the functional limitations of patients with COPD, since they present similar metabolic, ventilatory, cardiovascular and dyspnea responses.

The 6MWT is a test widely used to verify the physical capacity of patients with COPD. Several studies have shown the relationship between the 6MWT and peak of oxygen consumption (VO2) in the cardiopulmonary test which is considered a gold-standard test^12^ ^,^ ^37^ ^-^ ^41^. Another study examined the relationship between the 6MWT and other tests, which assessed different aspects of functional capacity, and found a relationship between the distance traveled and sit-to-stand activity (r=0.67), static balance (r=0.52) and gait velocity (r=-0.71)^42^. In addition, the 6MWT had its criterion validity determined and can be used to predict mortality^12^ ^,^ ^13^ ^,^ ^15^ ^,^ ^43^. All these features favour the use of the 6MWT as a comparison test for the construction validity of other functional tests. 

Considering the concurrent criterion validity for the 6MST, we observed a significant and strong positive correlation (r=0.734) between the performances in the test and the 6MWT. The relationship between these two tests has been studied by Machado et al.^20^ in 2008. These authors found no statistical difference between the physiological (HR and SpO_2_) and subjective (perceived exertion) variables studied and reported no correlation between the two tests; however, it is unclear if the performance in both tests was correlated. Note that, in the study, the height of the step was 14.5 cm, differing from the present study, in which the step used was 20 cm in height. Similarly, Schnaider and Karsten^19^ observed that the number of steps on the 6MST with free cadence showed a good correlation (r=0.706; p<0.001) with the 6MWT in critical, hospitalized patients with COPD. However, it is worth mentioning that these authors used a step of 15 cm in height by 40 cm deep and 60 cm wide and conducted only one test. The present study is the first to verify that the 6MST compared to the 6MWT, using the methodological steps described earlier, can determine reduced functional capacity resulting from COPD. It also showed that the 6MST correlated with the BODE prognostic index and with the FEV_1_ - although the correlation was weak - suggesting that the performance at the 6MST can be considered a marker of the severity of the disease.

Similarly, other studies have shown that the BODE prognosis index was associated with other methods of assessing functionality, such as the sit-to-stand in two minutes, the 6MWT performed on a treadmill^44^, the level of physical activity in daily life^45^ and limitations in activities of daily living^46^.

Predictive validity criterion was determined at T1 or T2, or solely at T1, with the 6MST, using the 6MWT as the gold-standard to classify individuals with COPD concerning their physical capacity. For both comparisons, the 6MST was shown to be accurate for some purposes according to the classification of Swets^33^. This result suggests that the use of this test as a criterion for indication for surgical interventions, a lung transplant for example, should be avoided and new studies should be conducted to check whether it is appropriate for this purpose.

The fact that T1 presented concurrent and predictive criterion validity suggests that there is no need for a familiarization test. It also indicates that it might be interesting to evaluate the test on a larger sample. Based on the cut-off of 78 steps, patients with similar or lower values can be classified as having a low physical capacity, suggesting the need for physical therapy intervention. However, it is important to consider that the present study had a lower than ideal sample to verify criterion validity, suggesting the need of future studies.

### Study limitations

The present study had the following limitations. It was not designed to verify predictive validity, since a larger sample size would be needed for this type of analysis. However, it should be emphasised that the study raised the hypothesis of a relationship between the 6MST and the prognosis of patients with COPD, which should be addressed in future studies.

## Conclusion

The performance observed on the 6MST was valid for determining low physical capacity in patients with COPD with a cut-off of 78 steps, whereas values below that represented patients having a poorer prognosis.
